# Analyzing the practice of medical humanistic care based on a social learning model: mapping the trajectory of the learning dynamic process from the learner’s perspective

**DOI:** 10.3389/fmed.2025.1601951

**Published:** 2025-06-11

**Authors:** Bilu Gu, Yiming Lv, Jiyu Zhu, Xiaoling Sun, Kun Li

**Affiliations:** ^1^Department of Outpatient, Nanjing Drum Tower Hospital, Affiliated Hospital of Medical School, Nanjing University, Nanjing, China; ^2^School of Nursing, Nanjing University of Chinese Medicine, Nanjing, China; ^3^Department of Medical, Nanjing Drum Tower Hospital, Affiliated Hospital of Medical School, Nanjing University, Nanjing, China

**Keywords:** nursing, master’s students, humanistic care, education, qualitative, clinical practice

## Abstract

**Background:**

Humanistic care is a good glue for the doctor-patient relationship, and it is a general trend to improve the practice of humanistic care.

**Methods:**

A narrative research method was used to conduct semi-structured interviews with 18 master’s degree nursing students from China who were in the clinical rotation stage, and the data were content analyzed and explored from the perspective of the learners who were learning about humanistic caring practices using the social learning theory model.

**Results:**

There is a triple tension structure in the practice of humanistic care: At the cognitive level, there is a knowledge-activity rupture, with learners showing theoretical clarity but practical confusion. At the environmental level, it is divided into the dual role of facilitating and inhibiting environments. “rewarding” environments included positive psychological attitudes of patients, caring-friendly departmental environment, perceptually rewarding mindfulness environment, and loving family environment. In contrast, “punishing” environments included patients’ irresponsible attitudes toward themselves, poor care experiences, inflexible management mechanisms, missing incentives. At the behavioral level, there is a dialectical game between constructive and alienating practices. “forward” behaviors included personalized care in the details, respect for patient autonomy, proactive communication and empathy, systemic support and teamwork. Conversely, “backward” behaviors included mechanized procedures and emotional detachment, disregard for privacy and dignity, systemic issues that exacerbate apathy.

**Conclusion:**

Based on the framework of social learning theory, this study constructs a learning trajectory model of humanistic care to explain the synergistic mechanism between cognitive dimension and environmental system and its two-way shaping of caring practice behavior. The study finds that there is a “black box” phenomenon in which the theory of humanistic care is clear but the practice of humanistic care is confusing in the cognitive dimension, and in the environmental dimension, there are systematic limitations in the traditional biomedical model. Based on the above two-dimensional analysis, this study proposes an optimization path combining cognitive explicit cultivation and environmental support system reconstruction, which points out the direction for breaking through the dilemma of humanistic care practice.

## 1 Introduction

In recent years, China has seen a high incidence of violence and injuries due to tensions between medical professionals and patients, and according to China’s Supreme People’s Court, a total of 159 medical crime cases have been reported between January 2019 and April 2020 alone (1). These acts of violence have had an immeasurably bad impact on both medical professionals and patients, as well as on the reform of healthcare services (2). This phenomenon is not only occurring in China, according to the World Health Organization, about 8–38% of medical professionals have been subjected to physical violence at some point in their careers (3). The frequency of violence is essentially a manifestation of tensions between medical professionals and patients, and several studies (4, 5) have pointed out that humanistic care can significantly improve the quality of clinical healthcare work, including the ability to significantly improve doctor-patient relationships, and enhance patient satisfaction, psychological wellbeing, and positive health outcomes. Nursing staff is the backbone of clinical medical work. According to Watson and Brewer (6), a preeminent American scholar in the field of humanistic care, the core and essence of nursing is humanistic care. WHO also identified patient-centered care as one of the five core competencies of nursing staff at an early stage. In previous studies, researchers have also elaborated on humanistic care in nursing (7) and extensively explained the importance of humanistic care in nursing (8), and researchers usually focus on different educational approaches and interventions on how to enhance humanistic care in nursing: Deng et al. (9) innovatively introduced a humanistic care digital storytelling course to enhance the humanistic care competence of nursing students in the intensive care unit (ICU). Xue et al. (10, 11) applied the Carolina Care Model to obstetrics and gynecology nurses to improve humanistic nursing practice. Huang et al. (12) employed a multidimensional humanistic approach to create a warm hospital experience in a women’s and children’s center. Previous studies (9–12) have found that humanistic care competencies are often related to the communication, emotional processing, and emotional intelligence of medical professionals, and the formation of these competencies is often the result of the interaction between the individual’s cognitive, behavioral, and environmental aspects (13). However, in the existing studies addressing humanistic care enhancement, researchers have taken a third-person perspective, and few researchers have explored the humanistic care learner perspective. This study breaks through the objectification research paradigm prevalent in the existing literature and innovatively selects Master of Nursing Studies (MNS) postgraduates as the research vehicle for practice epistemology, in response to the lack of subjective perspectives in the field of humanistic care education research. This group has both a practical knowledge system shaped by clinical habitus and a collegiate theoretical cognitive framework, and their dynamically evolving clinical practice trajectory provides a phenomenological window for observing contextualized learning in the community of practice. Based on the practitioner-oriented epistemological turn, this study deconstructs the mechanism of the relationship between cognition, environment and behavior based on social learning theory, and focuses on revealing the logic of the enhancement of practical ability in clinical practice, thus exploring the optimal path to improve the practice of humanistic care in medicine.

### 1.1 Theoretical foundations

Bandura put forward the social learning theory through the “Bobo doll experiment” (14), the theory focuses on the role of learning and self-regulation in triggering human behavior, and explores the influence of human cognition, behavior and environment and their interaction on human practical behavior. The results of the study found that the mechanism of “punishing” has a greater impact on the formation of individual behaviors, and that in experiments, seeing others being rewarded does not necessarily motivate us to imitate their behavior, while seeing others being punished greatly reduces our willingness to do so. The key tenet of the theory is that learning is a dynamic cognitive process that occurs in a social context and can occur through observing a behavior and the consequences of that behavior, but learning can also occur in the absence of observable behavioral change. The theory suggests that the learner’s cognition, behavior and environment all interact with each other, a process known as “reciprocal determinism.”

### 1.2 Purpose of the study

The purpose of this study is to understand the state of learners’ cognitive, behavioral and environmental aspects and the mechanism of the relationship between the three aspects during the learning process of humanistic caring practices and to further explore how to enhance the optimal path of learners’ humanistic caring practices based on Bandura’s social learning theory (14).

## 2 Materials and methods

### 2.1 Design

The study used a descriptive qualitative research design to conduct in-depth semi-structured interviews. Additionally, narratives related to humanistic care practices were collected to facilitate a comprehensive exploration of participants’ experiences with humanistic care in clinical settings.

### 2.2 Setting and participants

Conducted in China, this study recruited master’s degree nursing students from four tertiary hospitals in the Nanjing area to capture diverse experiences of humanistic care practices. Purposive sampling was used, with inclusion criteria consisting of: (1) master’s degree nursing students with at least two years of clinical experience, and (2) those currently in the clinical practice phase. Participants were recruited via the WeChat platform after a brief introduction to the study. Of the 20 initial respondents, 18 met the inclusion criteria and consented to participate.

### 2.3 Data collection

Ethical approval was obtained from the Medical Ethics Committee of Drum Tower Hospital, School of Medicine, Nanjing University (Grant No. 2024-409). Prior to the study, participants were provided with an informed consent form outlining the study’s purpose and assurances of confidentiality and anonymity. All data were restricted to the research team, and any identifying information from interviews was removed from transcripts and reports.

The first author conducted 18 semi-structured, face-to-face interviews between September 2024 and January 2025, following an interview guide (Table 1). Each interview was audio-recorded, with on-site note-taking and observation. To ensure participant comfort, interviews were held in quiet, private classrooms in a conversational manner, lasting 30–40 min. Audio recordings were transcribed verbatim on the same day, and participants’ narratives were organized using a first-person narrative structure (15). These narratives were then reviewed and adjusted based on participant feedback to ensure accuracy.

**TABLE 1:** Interview guide.

Interview guide	
Cognition	① What is your understanding of humanistic care in a healthcare setting? What does it entail?
Behavior	② Could you describe a memorable experience where you implemented humanistic care for a patient? How did you feel, and what impact did it have on you?
Environment	③ Can you describe a situation where you encountered difficulties in implementing humanistic care? How did you address these challenges, and what was the outcome?
	④ What factors in your work environment have supported your practice of humanistic care? What additional factors do you believe could enhance your skills in this area? Do you have any suggestions or comments?

### 2.4 Data analysis

Initially, the data underwent a preliminary round of coding using thematic analysis. Each participant’s responses were summarized into a single sentence and further distilled into keywords. Subsequently, codes with similar meanings and conceptual relevance were grouped into sub-themes. The sub-themes were then analyzed through frame analysis, guided by Bandura’s Social Learning Theory (14). Table 2 illustrates the main structure of the framework.

**TABLE 2:** Framework structure.

Module	Includes
Cognitive	Comprehensive
	Incomprehensive
Behavioral	Forward
	Backward
Environmental	“Rewarding”
	“Punishing”

Atlas.ti 9.1.3 was employed to store, organize, and code the data for this study. The interview data were subject to thorough quality checks and double-checked with participants prior to coding and analysis. The coding process began with independent coding by each author, followed by a group discussion to minimize potential biases arising from individual perspectives.

## 3 Results

Among the 18 participants, 16 were employed at a tertiary general hospital, while 2 worked at a tertiary specialized dental hospital. All participants possessed an average of over three years of clinical experience and had rotated through more than 10 departments.

The following paragraphs first provide an in-depth description of the learners’ real-life experiences in exploring humanistic care practices from three dimensions: cognition, environment, and behavior. We then describe the learning trajectory in which these three dimensions interact with each other.

### 3.1 Cognition

This section examines the participants’ perceptions of humanistic care at two levels: theoretical and practical, the table summarizing participant quotes as shown in Table 3.

**TABLE 3:** Cognitive level participant quote summary table.

Theme	Module	Element	Participant quotes
Cognition	Theoretical level	Participants demonstrated a clear understanding of the importance and components of humanistic care, with a particular focus on patients’ psychological needs.	“I think humanistic care should not only pay attention to the patient’s physical health, but also to the patient’s mental health, is in addition to the basic work (injections, medications, blood draws) in addition to the spontaneous to do some caring behaviors, like I will probably comfort the patient, to do the operation process to pay more attention to the patient.” (Participant 2)
		Learners emphasized the attributes of a highly humanistic healthcare professional. Among these, technical competence was identified as foundational for building patient trust and a prerequisite for effective humanistic care	“If you can’t perform basic technical tasks properly, patients won’t trust you, making it very difficult to implement humanistic care.” (Participant 18)
	Practical level	When patients face complex life and death situations, participants empathize and offer proactive help perceived as an ulterior motive, a distrust that leaves them frustrated and confused about how to practice humanistic care.	“That patient was not resuscitated and I really couldn’t hold back my tears at that time, but my teacher told me that I couldn’t cry or else if the family members of patients saw it they might think it was due to my faulty work. I was suddenly sadder and felt helpless, I didn’t know why the trust between people was so fragile and I didn’t know what to do for the best.” (Participant 1)
		The first encounter with a patient is the best opportunity to build trust between nurse and patient.	“For a patient who has just been admitted to the hospital, it is important to have humanistic care in the process of measuring vital signs to help the patient quickly integrate into this unfamiliar environment in the hospital, which is the best time to gain the patient’s trust because the first sense of security that the patient comes to the hospital is given by you.” (Participant 3)
		A paradox exists in nurse-patient interactions: the desire to provide high-quality care often conflicts with the pressure to maintain efficiency.	“In fact, there is a situation in which, because I was very considerate to a patient before, this patient would keep coming to pester me, resulting in me not being able to get my work done. In that case, it makes me not dare to be too enthusiastic and considerate to the patient, because if once this opening is made, this patient may keep pestering me and affect my normal work. Plus, the nurse-patient relationship is still quite tense nowadays, so if I always refuse when a patient comes to initiate help, it may also bring some trouble.” (Participant 5)

#### 3.1.1 Theory: qualities required for humanistic medical professionals

At the theoretical level, participants demonstrated a clear understanding of the importance and components of humanistic care, with a particular focus on patients’ psychological needs.

“I think humanistic care should not only pay attention to the patient’s physical health, but also to the patient’s mental health, is in addition to the basic work (injections, medications, blood draws) in addition to the spontaneous to do some caring behaviors, like I will probably comfort the patient, to do the operation process to pay more attention to the patient.” (Participant 2)

**FIGURE 1:**
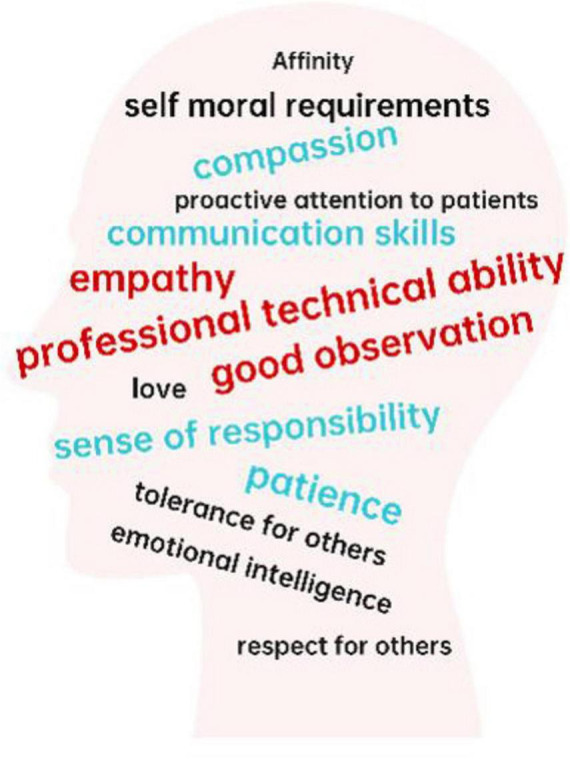
Frequency of mentioned attributes (Red: > 5 mentions, Blue: 3–5 mentions, Black: 1–2 mentions).

Participants emphasized 14 key attributes of highly humanistic medical professionals, listed in descending order of frequency: professional technical ability, empathy, good observation, patience, communication skills, compassion, sense of responsibility, tolerance for others, emotional intelligence, affinity, emotional intelligence, respect for others, proactive attention to patients, self moral requirements. These attributes were visualized in a word cloud based on their frequency of mention (Figure 1). Among these, technical competence was identified as foundational for building patient trust and a prerequisite for effective humanistic care:

“If you can’t perform basic technical tasks properly, patients won’t trust you, making it very difficult to implement humanistic care.” (Participant 18)

#### 3.1.2 Practice: humanistic care specific practice confusion

Despite their strong theoretical foundation, participants often experience confusion and uncertainty in real-world humanistic care practice, primarily due to the complex nature of doctor-patient interactions.

When patients face complex life and death situations, participants empathize and offer proactive help perceived as an ulterior motive, a distrust that leaves them frustrated and confused about how to practice humanistic care.

“That patient was not resuscitated and I really couldn’t hold back my tears at that time, but my teacher told me that I couldn’t cry or else if the family members of patients saw it they might think it was due to my faulty work. I was suddenly sadder and felt helpless, I didn’t know why the trust between people was so fragile and I didn’t know what to do for the best.” (Participant 1)

The first encounter with a patient is the best opportunity to build trust between nurse and patient.

“For a patient who has just been admitted to the hospital, it is important to have humanistic care in the process of measuring vital signs to help the patient quickly integrate into this unfamiliar environment in the hospital, which is the best time to gain the patient’s trust because the first sense of security that the patient comes to the hospital is given by you.” (Participant 3)

However, patient trust can sometimes present a double-edged sword for participants. A paradox exists in nurse-patient interactions: the desire to provide high-quality care often conflicts with the pressure to maintain efficiency.

“In fact, there is a situation in which, because I was very considerate to a patient before, this patient would keep coming to pester me, resulting in me not being able to get my work done. In that case, it makes me not dare to be too enthusiastic and considerate to the patient, because if once this opening is made, this patient may keep pestering me and affect my normal work. Plus, the nurse-patient relationship is still quite tense nowadays, so if I always refuse when a patient comes to initiate help, it may also bring some trouble.” (Participant 5)

### 3.2 Environment

The environments described by the participants can be classified into two categories: “rewarding” environments and “punishing” environments, the table summarizing participant quotes as shown in Table 4.

**TABLE 4:** Environmental level participant quote summary table.

Theme	Module	Element	Participant quotes
Environment	“rewarding” environments	Positive psychological attitudes of patients	“I encountered an 80-year-old grandmother with oral cancer in the ward. Her family provided constant care and encouragement. Unlike other elderly patients, she maintained a positive attitude, actively engaging with our guidance. Despite her weakened condition—requiring enteral nutrition and experiencing severe gastrointestinal reactions—she insisted on daily activities to aid her recovery. Her resilience impressed me, prompting me to offer additional attention and support.” (Participant 2) “If a patient explicitly seeks our help, we generally don’t refuse, provided the request is reasonable.” (Participant 12)
		Caring-friendly departmental environment	“In terms of the ward environment, if the ward environment could be more comfortable and welcoming, both the patient and us would be in a good mood, which is conducive to our caring mood.” (Participant 3) “If leaders can understand the hardship of nurses’ work, care for nurses, and take the initiative to help nurses, this can have a particularly strong impact on the atmosphere of the unit and the nurses’ wellbeing at work. How can nurses be expected to care for others when they themselves are not cared for and are having a bad time.” (Participant 4) “It would be nice to have less workload in the department, it’s so busy there’s really no way to be attentive to the emotions and needs of the patients.” (Participant 16) “In fact, some operations are actually different from hospital to hospital, and sometimes patients raise questions, but with such a busy clinical schedule, there is no way to explain every point to the patient. But then the patient will have more questions and it will lead to their trust to decrease.” (Participant 3) “For example, when I went to the cardiology department before, I found that their head nurse would be particularly concerned about each patient and really kept the patient’s needs in mind to implement them, and so did the nurses in the department, so I was infected then, and I would go and take the initiative to implement humanistic care, and I was able to learn how to implement humanistic care from them as well. But then I went to another department and the people in that department were very indifferent and I felt it was difficult for me to implement humanistic care alone in that environment.” (Participant 7) “Between colleagues, if you do this humanistic care and people think you are doing a good job and give you encouragement, you feel that this is meaningful.” (Participant 8)
		Perceptually rewarding mindfulness environment	“I feel that we all get a sense of reward in human care and a sense of fulfilment, being able to see that a person has been helped and guided by you and they are gradually becoming more resilient and recovering both physically and mentally.” (Participant 7) “Their psychological support is gradually stronger, and some patients even send us flags to express their gratitude, we feel affirmed, and that happy feeling is unique.” (Participant 9) “From the indifference at the beginning, and then the patient was really touched, I could read it in her eyes, and at that time, I was also very touched, and for the first time I thought, oh my God, I saved her, and that kind of touched me, and I don’t think I’ll ever forget it.” (Participant 15)
		Loving family environment	“When I was on placement, I encountered a lot of difficulties and at that point I thought about just treating this as just a job and giving up on humanistic care. But my family gave me a lot of support and that’s when I thought I mustn’t let them down, I can do better.” (Participant 2) “At that time, my grandmother was sick and I was really worried about her, and I noticed a lot of her subtle needs and later learnt to think differently when dealing with patients.” (Participant 14)
	“punishing” environments	Patients’ irresponsible attitudes toward themselves	“He had been suffering from the disease for too long, and when dealing with patients like that, they all have a characteristic that they won’t do anything no matter what we say in terms of instructions. At that time he was lying in bed all day and prone to pressure sores, I told him to go for more activity to promote gastrointestinal function so that he could consider stopping the enteral nutritional support because he was already having some uncomfortable reactions like nausea and vomiting. But he didn’t bother with my advice, and he was in a state of personal numbness and distrust. I felt powerless and even disappointed.” (Participant 5)
		Poor care experiences	“I have found that when I repeatedly care for my patients during treatment, some patients appreciate it. But some patients would instead doubt my job and think that I am not doing a good job, otherwise why would I care for them so much.” (Participant 11) “A patient’s family, all of a sudden he threw his hand and slapped me, he yelled at me for meddling in my business, I felt that if I hadn’t been acting out of conscientiousness I wouldn’t have suffered all this, I was so disappointed and it seemed like after that I would rarely take the initiative to do anything related to humanistic care.” (Participant 1) “That patient was giving me all the abusive words but I couldn’t say a word back because I’m wearing this white coat and I’m a medical worker, but should I be a medical worker and I should have to bear this?” (Participant 12) “I was especially helpless at that point, holding back tears and looking to my lead teacher. She seemed like she was suddenly very busy and didn’t go to see me.” (Participant 10) “Because the internship phase is actually a nurse’s first exposure to the clinic and the most vulnerable time in their career, if anything, this stage is full of negative experiences, it could lead to this nurse not giving more emotion, care to the patient, and I think it’s also a protective mechanism for the person.” (Participant 4)
		Inflexible management mechanisms	“From the management level, hospitals now include humanistic care in their assessment criteria, and we should strive to be objective in these criteria. However, we now take satisfaction as the criterion for judgment, which is actually subjective. If a nurse encounters an extreme patient, the patient will subjectively judge the nurse poorly and fail to cooperate, resulting in the failure of humanistic care. I think that special cases like this should also be dealt with on an *ad hoc* basis by adopting a more objective and scientific way of judging, for example, other patients in the ward and other nurses in the same department can be approached to make a judgement on the matter and give medical professionals a channel to lodge complaints.” (Participant 6)
		Missing incentives	“There aren’t really any specific incentives for you to do that in hospitals these days, and who doesn’t want to be a good person and care more about their patients? But sometimes it’s still quite frustrating to realize that there’s not much difference between doing it and not doing it.” (Participant 2) “I don’t feel there is any clear benefit to my personal career development or title advancement.” (Participant 13)

#### 3.2.1 “rewarding” environments

Four primary themes emerged under this subtheme: (1) positive psychological attitudes of patients, (2) caring-friendly departmental environment, (3) perceptually rewarding mindfulness environment, (4) loving family environment.

(1) Positive psychological attitudes of patients

Continuous encouragement from family members creates a “psychological safety net” that alleviates patients’ anxiety related to disease uncertainty and fosters a positive psychological attitude. This positive engagement by patients can, in turn, influence healthcare professionals, enhancing their sense of efficacy and motivating them to provide proactive care.

“I encountered an 80-year-old grandmother with oral cancer in the ward. Her family provided constant care and encouragement. Unlike other elderly patients, she maintained a positive attitude, actively engaging with our guidance. Despite her weakened condition—requiring enteral nutrition and experiencing severe gastrointestinal reactions—she insisted on daily activities to aid her recovery. Her resilience impressed me, prompting me to offer additional attention and support.” (Participant 2)

“If a patient explicitly seeks our help, we generally don’t refuse, provided the request is reasonable.” (Participant 12)

(2) Caring-friendly departmental environment

A comfortable and welcoming physical environment promotes a caring atmosphere.

“In terms of the ward environment, if the ward environment could be more comfortable and welcoming, both the patient and us would be in a good mood, which is conducive to our caring mood.” (Participant 3)

A humanistic environment conducive to caring depends on a good leader. The leader’s humanistic caring quality has a great influence on the medical professionals’ humanistic caring initiative. The leader’s care and consideration for the medical professionals directly affects the medical professionals’ sense of wellbeing at work and directly determines whether they can have the ability to care for others.

“If leaders can understand the hardship of nurses’ work, care for nurses, and take the initiative to help nurses, this can have a particularly strong impact on the atmosphere of the unit and the nurses’ wellbeing at work. How can nurses be expected to care for others when they themselves are not cared for and are having a bad time.” (Participant 4)

The heavy clinical workload often means that the implementation of humanistic care by medical professionals is not a question of willingness, but a matter of feasibility. This dilemma highlights the need for leaders to optimize work distribution and enhance efficiency.

“It would be nice to have less workload in the department, it’s so busy there’s really no way to be attentive to the emotions and needs of the patients.” (Participant 16)

“In fact, some operations are actually different from hospital to hospital, and sometimes patients raise questions, but with such a busy clinical schedule, there is no way to explain every point to the patient. But then the patient will have more questions and it will lead to their trust to decrease.” (Participant 3)

Moreover, the humanistic behaviors exhibited by leaders and colleagues within the department, as well as the recognition of humanistic care’s value, subtly influence individuals’ willingness and actions to implement humanistic care.

“For example, when I went to the cardiology department before, I found that their head nurse would be particularly concerned about each patient and really kept the patient’s needs in mind to implement them, and so did the nurses in the department, so I was infected then, and I would go and take the initiative to implement humanistic care, and I was able to learn how to implement humanistic care from them as well. But then I went to another department and the people in that department were very indifferent and I felt it was difficult for me to implement humanistic care alone in that environment.” (Participant 7)

“Between colleagues, if you do this humanistic care and people think you are doing a good job and give you encouragement, you feel that this is meaningful.” (Participant 8)

(3) Perceptually rewarding mindfulness environment

The practice of humanistic care generates intrinsic satisfaction, stemming from the recognition of one’s own value and the realization that one’s actions can positively impact others.

“I feel that we all get a sense of reward in human care and a sense of fulfilment, being able to see that a person has been helped and guided by you and they are gradually becoming more resilient and recovering both physically and mentally.” (Participant 7)

Moreover, the full recognition by patients of the value of healthcare workers’ efforts can nourish their spirit.

“Their psychological support is gradually stronger, and some patients even send us flags to express their gratitude, we feel affirmed, and that happy feeling is unique.” (Participant 9)

Concurrently, observing positive changes in patients reinforces self-value identification, creating a positive feedback loop that motivates healthcare workers to continue practicing humanistic care.

“From the indifference at the beginning, and then the patient was really touched, I could read it in her eyes, and at that time, I was also very touched, and for the first time I thought, oh my God, I saved her, and that kind of touched me, and I don’t think I’ll ever forget it.” (Participant 15)

(4) Loving family environment

Within the theoretical and practical framework of humanistic care, this process is fundamentally a dynamic interplay of love and interaction. For participants, the emotional support and care provided by their families serve as the initial source of love, offering an essential internal motivation for cognitive development, emotional maturation, and social adaptation. This support is deeply embedded in their personal growth trajectories, playing a foundational and enduring role in shaping their personalities and learning capabilities.

“When I was on placement, I encountered a lot of difficulties and at that point I thought about just treating this as just a job and giving up on humanistic care. But my family gave me a lot of support and that’s when I thought I mustn’t let them down, I can do better.” (Participant 2)

“At that time, my grandmother was sick and I was really worried about her, and I noticed a lot of her subtle needs and later learnt to think differently when dealing with patients.” (Participant 14)

#### 3.2.2 “punishing” environment

Four primary themes emerged under this subtheme: (1) Patients’ irresponsible attitudes toward themselves, (2) Poor care experiences, (3) Inflexible management mechanisms, (4) Missing incentives.

(1) Patients’ irresponsible attitude toward themselves

Due to the psychological trauma of long-term illness on the one hand, and the cognitive limitations of patients on the other hand, it is easy for them to experience communication difficulties, distrust of others, and a partial abandonment of their own health. In the face of patients’ negative attitudes, medical personnel often feel professional frustration, psychological burden and inability to cope.

“He had been suffering from the disease for too long, and when dealing with patients like that, they all have a characteristic that they won’t do anything no matter what we say in terms of instructions. At that time he was lying in bed all day and prone to pressure sores, I told him to go for more activity to promote gastrointestinal function so that he could consider stopping the enteral nutritional support because he was already having some uncomfortable reactions like nausea and vomiting. But he didn’t bother with my advice, and he was in a state of personal numbness and distrust. I felt powerless and even disappointed.” (Participant 5)

(2) Poor care experience

Due to the specificity of patients’ individual states and the complexity of their psychological states, even if medical staff care for patients with goodwill and a sense of responsibility, the result may be two very different types of feedback, one of gratitude and the other of skepticism.

“I have found that when I repeatedly care for my patients during treatment, some patients appreciate it. But some patients would instead doubt my job and think that I am not doing a good job, otherwise why would I care for them so much.” (Participant 11)

When mistrust from patients or their families spreads further, it may trigger violent incidents. Violent incidents not only inflict physical harm on medical professionals but also cause significant psychological trauma. These psychological effects can alter individual work attitudes, potentially lead to burnout, and cause medical professionals to lose enthusiasm and motivation for their jobs, which may even negatively impact the quality of care across the medical sector.

“A patient’s family, all of a sudden he threw his hand and slapped me, he yelled at me for meddling in my business, I felt that if I hadn’t been acting out of conscientiousness I wouldn’t have suffered all this, I was so disappointed and it seemed like after that I would rarely take the initiative to do anything related to humanistic care.” (Participant 1)

Within medical teams, there is often a lack of timely and effective support systems for members who suffer injuries.

“That patient was giving me all the abusive words but I couldn’t say a word back because I’m wearing this white coat and I’m a medical worker, but should I be a medical worker and I should have to bear this?” (Participant 12)

Additionally, the avoidance behavior of colleagues can exacerbate the victim’s sense of isolation and further intensify their psychological trauma.

“I was especially helpless at that point, holding back tears and looking to my lead teacher. She seemed like she was suddenly very busy and didn’t go to see me.” (Participant 10)

Notably, the internship phase is a critical transition period for nursing professionals as they integrate into clinical practice for the first time. This period is also marked by heightened vulnerability in their occupational psychology. If nursing interns experience a series of negative events, such as doctor-patient conflicts, work-related stress, or difficulties with professional identity, it is highly likely to trigger a psychological defense mechanism. This mechanism can significantly reduce their subsequent willingness to provide humanistic care to patients.

“Because the internship phase is actually a nurse’s first exposure to the clinic and the most vulnerable time in their career, if anything, this stage is full of negative experiences, it could lead to this nurse not giving more emotion, care to the patient, and I think it’s also a protective mechanism for the person.” (Participant 4)

(3) Inflexible management mechanisms

Currently, the quantitative assessment of humanistic care faces significant challenges. Using satisfaction as a sole criterion for evaluation has inherent limitations. Medical professionals have called for the establishment of multiple channels for assessment and feedback.

“From the management level, hospitals now include humanistic care in their assessment criteria, and we should strive to be objective in these criteria. However, we now take satisfaction as the criterion for judgment, which is actually subjective. If a nurse encounters an extreme patient, the patient will subjectively judge the nurse poorly and fail to cooperate, resulting in the failure of humanistic care. I think that special cases like this should also be dealt with on an *ad hoc* basis by adopting a more objective and scientific way of judging, for example, other patients in the ward and other nurses in the same department can be approached to make a judgement on the matter and give medical professionals a channel to lodge complaints.” (Participant 6)

(4) Missing incentive mechanisms

participants’ intrinsic motivation and willingness to provide proactive care were evident. However, the absence of incentives meant that their extra efforts in practice often went unrewarded with significant positive feedback. This prolonged discrepancy between their internal drive and external reality can erode enthusiasm and motivation, ultimately undermining work engagement and professional fulfillment.

“There aren’t really any specific incentives for you to do that in hospitals these days, and who doesn’t want to be a good person and care more about their patients? But sometimes it’s still quite frustrating to realize that there’s not much difference between doing it and not doing it.” (Participant 2)

“I don’t feel there is any clear benefit to my personal career development or title advancement.” (Participant 13)

### 3.3 Behavior

Participants’ humanistic behaviors can be categorized as “forward” and “backward,” the table summarizing participant quotes as shown in Table 5.

**TABLE 5:** Behavioral level participant quote summary table.

Theme	Module	Element	Participant quotes
Behavior	“forward”	(1) Personalized care in the details	“Addressing the patient by name rather than bed number is also a sign of respect for the patient.” (Participant 9) “That diabetic patient, I am particularly patient with his health promotion every time, and the other day I noticed that his injection site was already purple and hard, so I put a hot compress on him.” (Participant 4)
		(2) Respect for patient autonomy	“For the choice of treatment options, sometimes the patient just doesn’t take it, and we can only respect him, he has that right, but we will talk about the pros and cons and do what we have to do.” (Participant 7) “Like care plans and stuff, we also communicate with him to see what he wants and what he thinks.” (Participant 11)
		(3) Proactive communication and empathy	“Like when a patient chooses a prosthetic breast, I will give her certain advice taking into account her financial situation.” (Participant 18) “For less educated patients, I am using a more grounded approach, avoiding jargon and trying to communicate and explain to the patient as plainly as possible, if they still don’t understand, I choose to communicate with the patient’s family members who are more educated.” (Participant 14)
		(4) Systemic support and teamwork	“I think the model of multidisciplinary holistic management in my hospital just allows for a more detailed focus on patient needs because it’s individualizing the patient and focusing more on all aspects of a patient.” (Participant 12)
	“backward”	(1) Mechanized procedures and emotional detachment	“I feel that this leads to a processualization of care (e.g., regular turning, injections) and a lack of proactive observation of the patient’s needs.” (Participant 7) “That patient was crying all the time, but her condition was not serious, so I didn’t pay attention to her.” (Participant 11)
		(2) Neglect of privacy and dignity	“When we discussed the condition of the patient in bed 1 during the morning rounds, probably because the patient in the next bed could hear us, the patient in bed 1 acted very formal and didn’t talk about her needs.” (Participant 13) “She was a long term bedridden patient and because the patient in the next bed was not there at the time, we left the area uncovered when we looked at it and we didn’t realize that she was acting disturbed.” (Participant 4)
		(3) Systemic issues fueling apathy	“I told the patient that I would come over later and I wanted to get busy and go over there, but the work in my hand never stopped.” (Participant 1) “Being questioned by the patient in this manner was very disappointing. Moving forward, I would like to minimize any additional interactions with the patient to avoid unnecessary complications.” (Participant 15)

#### 3.3.1 “Forward”

Four primary themes emerged under this sub-theme: (1) personalized care in the details, (2) respect for patient autonomy, (3) proactive communication and empathy, (4) systemic support and teamwork.

(1) Personalized care in the details

Patient-centered, humanistic practice based on individual differences and needs:

“Addressing the patient by name rather than bed number is also a sign of respect for the patient.” (Participant 9)

“That diabetic patient, I am particularly patient with his health promotion every time, and the other day I noticed that his injection site was already purple and hard, so I put a hot compress on him.” (Participant 4)

(2) Respect for patient autonomy

The spirit of modern medical ethics and the concept of humanistic care are employed to protect patients’ rights to autonomy and participation:

“For the choice of treatment options, sometimes the patient just doesn’t take it, and we can only respect him, he has that right, but we will talk about the pros and cons and do what we have to do.” (Participant 7)

“Like care plans and stuff, we also communicate with him to see what he wants and what he thinks.” (Participant 11)

(3) Proactive communication and empathy

Communication patterns are adapted flexibly to accommodate differences in patient contexts:

“Like when a patient chooses a prosthetic breast, I will give her certain advice taking into account her financial situation.” (Participant 18)

“For less educated patients, I am using a more grounded approach, avoiding jargon and trying to communicate and explain to the patient as plainly as possible, if they still don’t understand, I choose to communicate with the patient’s family members who are more educated.” (Participant 14)

(4) Systemic support and teamwork

Team-based, individualized patient management is central to this approach, encouraging multidisciplinary team members to meticulously address all aspects of patient needs.

“I think the model of multidisciplinary holistic management in my hospital just allows for a more detailed focus on patient needs because it’s individualizing the patient and focusing more on all aspects of a patient.” (Participant 12)

#### 3.3.2 “Backward”

Three primary themes emerged under this sub-theme: (1) mechanized procedures and emotional detachment, (2) disregard for privacy and dignity, (3) systemic issues that exacerbate apathy.

(1) Mechanized procedures and emotional detachment

Neglecting patients’ emotional and potentially non-conditional needs:

“I feel that this leads to a processualization of care (e.g., regular turning, injections) and a lack of proactive observation of the patient’s needs.” (Participant 7)

“That patient was crying all the time, but her condition was not serious, so I didn’t pay attention to her.” (Participant 11)

(2) Neglect of privacy and dignity

There is an urgent need to optimize the process of privacy protection to suit the physical and psychological needs of patients:

“When we discussed the condition of the patient in bed 1 during the morning rounds, probably because the patient in the next bed could hear us, the patient in bed 1 acted very formal and didn’t talk about her needs.” (Participant 13)

“She was a long term bedridden patient and because the patient in the next bed was not there at the time, we left the area uncovered when we looked at it and we didn’t realize that she was acting disturbed.” (Participant 4)

(3) Systemic issues fueling apathy

Trust dilemmas and shifts in healthcare professionals’ self-protection strategies triggered by factors such as scheduling and communication in nurse-patient interactions:

“I told the patient that I would come over later and I wanted to get busy and go over there, but the work in my hand never stopped.” (Participant 1)

“Being questioned by the patient in this manner was very disappointing. Moving forward, I would like to minimize any additional interactions with the patient to avoid unnecessary complications.” (Participant 15)

### 3.4 Learning trajectories

**FIGURE 2:**
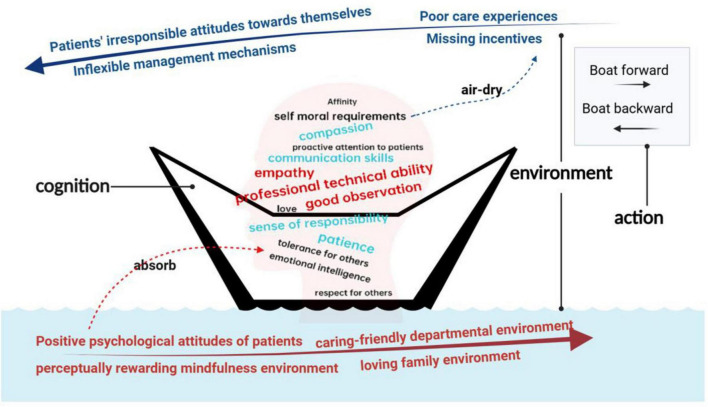
Trajectory map.

In summary of the results, mapping the learners’ learning trajectories based on cognitive, environmental and behavioral aspects, as shown in Figure 2, we found that there are a triple tension structure in humanistic care practice: These trajectories can be conceptualized as a “boat against the current.” Specifically, “cognition” represents the inherent characteristics of the human subject, akin to the role of a “sponge boat.” The “environment” is divided into “punishing” and “rewarding” factors. The “punishing” environment acts as a headwind, while the “rewarding” environment functions as a favorable current. “Behavior” corresponds to the ultimate direction of the boat.

The diagram depicts the learning path of humanistic care for learners: human as a subject, just like a sponge, cognition is affected by the environment, a good environment (“rewarding”) can be like a sponge sucking up water to enhance human cognition, which can be internalized into excellent traits; while a bad environment (“punishing”) is like the wind that hinders the ship’s progress while drying up the water of the sponge, thus weakening the excellent traits.

## 4 Discussion

Our analysis of the learners’ narratives elucidates the cognitive, environmental, and behavioral dimensions of their learning experiences in humanistic care, ultimately mapping the learning trajectories based on these three aspects.

The results of the learners’ perceptions showed a great contrast between those who showed theoretical clarity and those who were very confused in practice. Research indicates that while 82% of medical professionals endorse the concept of humanism, only 26% can articulate a standardized implementation process (16). Currently, humanistic care confronts a “black box” dilemma: its theoretical framework remains largely philosophical, lacking quantifiable behavioral guidelines and tools to assess effectiveness.

The environmental analysis shows that hospitals, patients, and personal growth environments collectively shape the cultivation of humanistic care. Maintaining a “rewarding” environment while addressing the “punishing” environment is crucial. The four major factors in the “punishing” environment highlight systemic limitations of the traditional biomedical model:(1) “Patients’ irresponsible attitudes toward themselves” signal the need for health empowerment; (2) “Poor care experiences” underscore the necessity for relational reshaping between medical professionals and patients; (3) “Inflexible management mechanisms” point to the need for organizational change in hospitals; and (4) “Missing incentives” highlight the necessity for incentive system design in medical organizations.

### 4.1 Recommendations

At the cognitive level, to address the “black box” dilemma of humanistic care, researchers must actively develop behavioral guidelines for caring practices, while educators should innovate teaching mechanisms in nursing education.

Utilizing the GRADE System, researchers can evaluate the effectiveness of caring measures to create “humanistic evidence-based guidelines.” This approach transforms the vague concept of caring into an observable, measurable, and improvable practice system, ultimately achieving the integration of knowledge and practice, encapsulated by the principle of “having the concept in mind and the tools in hand.”

Innovations in nursing education should focus on curriculum design and teaching methods. For nursing students who have not yet entered clinical practice, increasing the proportion of practical courses (17) and employing case-based teaching methods can immerse learners in real-world clinical scenarios (18), enhancing their understanding of patients’ emotions and needs. Additionally, incorporating multicultural nursing perspectives and introducing subjects such as psychology and sociology can broaden students’ horizons and deepen their understanding of patients’ psychological and social backgrounds. For nursing students entering clinical practice, emphasis should be placed on practical guidance and feedback. Students should be encouraged to reflect on their experiences through journaling and regular group discussions to foster continuous improvement (19).

At the environmental level, the construction of caring healthcare ecosystems is necessary to address the systemic limitations of the traditional biomedical model, as elaborated below in the context of the “rewarding” and “punishing” environments identified in the study.

The “rewarding” environment for humanistic care is the result of a synergistic interaction among three key components: the hospital (caring-friendly departmental environment), the patient (positive psychological attitudes of patients), and the individual (perceptually rewarding mindfulness environment and loving family environment). For hospitals, the development of caring healthcare ecosystems should focus on two main areas: hospital space reconstruction and humanistic care capacity building. In terms of hospital space reconstruction, “healing space design” (20) can be implemented to create a caring physical environment that enhances patients’ emotional experiences, particularly for those with emotional disorders. Additionally, visual decision support tools can be introduced to establish a “healthcare-patient co-healing space,” such as MediCoSpace by van der Linden et al. (21), which provides more comprehensive and personalized treatment options for patients. Regarding humanistic care capacity building, empathetic communication skills should be integrated into mandatory continuing education courses for healthcare professionals (22). Researchers can also refer to the “Humanistic Care Leadership Programme” by Saab et al. (23) to cultivate caring leaders and enhance medical professionals’ enthusiasm for humanistic care practice. For patients, it is crucial to build a positive psychological and emotional support system. Given that family support significantly improves patients’ psychological positivity and treatment compliance (24), effective home care models should be implemented. These include the use of “non-violent communication techniques” (25) to foster positive communication between patients and their families, and the implementation of a “family care log” (26) to document patients’ emotional changes and family interactions. Regular feedback from healthcare professionals is essential in this process. For individuals, a professional psychological capital training system should be established. Research indicates (27) that positive thinking moments can significantly reduce emotional exhaustion among medical professionals. Therefore, “clinical positive thinking moments” could be implemented before shifts to enhance workers’ psychological resilience. Moreover, neurological studies show that a sense of ceremony can activate the brain’s reward circuit (28). Hospitals could thus develop cultural mechanisms to enhance medical professionals’ psychological capital, such as ceremonies that anchor meaningful values: for example, an “initial ceremony” to revisit the Hippocratic Oath and a “shimmering moment” to share examples of humanistic care.

In the “punishing” environment of humanistic care, the “patients’ irresponsible attitude toward themselves” suggests that we should cultivate patients’ awareness of their health responsibilities and establish effective psychological support for them. Narrative interventions (10) can be used by medical professionals to improve patients’ self-efficacy by making them intuitively perceive the consequences of interventions. The “poor care experience” highlights the significant impact of the internship period on medical professionals. It suggests the need for an effective and feasible support system to address the negative experiences of medical professionals. Hospitals can establish “energy resupply stations,” such as the Mayo Clinic Employee Support Center, which provides immediate recovery facilities like 10-min rapid meditation pods and counseling robots (29). Two additional management-level issues in “punishing” environments are “inflexible management mechanisms” and “missing incentives.” According to Complexity Adaptive System (CAS) theory, local innovation is three times more successful than global reform (30). Management flexibility requires reserving space for dynamic policy experimentation, allowing departments to break through existing systems within a limited scope and rapidly disseminate successful experiences. An effective incentive mechanism for medical professionals to practice humanistic care can be implemented through “Time Banking” (31). In the study by Ng et al. (32), “Time Banking” was applied to elderly services, where volunteers exchanged their time for personal time credits for providing services to the elderly. These credits could be used for personal time exchanges when necessary, forming a virtuous cycle of enhancing social capital to maintain service exchange. Based on this, hospitals can implement a similar method by awarding humanistic points to medical professionals who practice humanistic care. A “care honor ladder” can be created to foster a sense of competition, and accumulated humanistic points can be exchanged for further training opportunities or qualifications, which can be used to compete for management positions.

## 5 Limitations

This study developed an interview framework grounded in Social Learning Theory, presenting the learning process of humanistic care from the learners’ perspective through narrative methods. It captured the dynamic nature of humanistic care learning and mapped the trajectory of interactions among cognitive, environmental, and behavioral dimensions. This approach revealed the “black box” dilemma of humanistic care at the cognitive level and the systemic limitations of the traditional biomedical model at the environmental level. However, while the analytical framework clarifies our findings, it may also act as a filter, potentially limiting our perspective.

## 6 Conclusion

Our research indicates that learners’ cognition is constantly changing based on innate inherent traits influenced by the environment, and that both cognition and environment together determine behavior. Additionally, the study highlights an often-overlooked aspect of the cognitive dimension of humanistic care: the “black box” dilemma. Resolving this dilemma could advance medical humanities from philosophical discourse to clinical practice. Future research should focus on developing a comprehensive list of caring behaviors across various humanistic care scenarios, as well as quantitative tools to evaluate their effectiveness. On the environmental level, the “rewarding” environment underscores the need to enhance the nurturing environment of humanistic care within hospitals, among patients, and for individuals. Conversely, the “punishing” environment highlights the necessity to dismantle the systemic limitations of the traditional biomedical model and construct a caring healthcare ecosystem. This study verifies the synergistic evolutionary mechanism of “cognition-environment-behavior” within the framework of social cognitive theory, and finds that learners’ cognition is constantly changing based on innate traits and influenced by the environment, and that cognition and the environment together determine behavior. At the cognitive level, the study reveals an aspect of humanistic care that is often overlooked: the “black box” dilemma of theoretical clarity and practical confusion. The solution to this dilemma is to move the medical humanities from philosophical discourse to clinical science, and in the future, researchers will need to continue to develop caring behavior inventories for different scenarios of humanistic caring, as well as quantitative tools for evaluating the effectiveness. At the environmental level, the “rewarding” environment suggests that we should improve the nurturing environment of humanistic care with a facilitating ecosystem (hospital-patient-individual triad), while the “punishing” environment suggests that we should break the systemic limitations of the traditional biomedical model and build a caring medical ecosystem.

## Data Availability

The data for this study are not publicly available due to privacy concerns. However, the data are available from the corresponding authors upon reasonable request. Requests for data will be considered on an individual basis, ensuring that any sharing complies with the ethical guidelines and privacy protections agreed upon by the participants. Requests to access the datasets should be directed to XS, sunxy081101@126.com.
